# Evaluation of Coronavirus Disease 2019 Burnout Syndrome Among Healthcare Workers in Taizhou, China

**DOI:** 10.3389/ijph.2023.1605539

**Published:** 2023-04-05

**Authors:** Tao-Hsin Tung, Yu-Pei Yang, Mei-Xian Zhang, Hai-Xiao Chen, Shuang-Jun Pan

**Affiliations:** ^1^ Evidence-Based Medicine Center, Taizhou Hospital of Zhejiang Province Affiliated to Wenzhou Medical University, Linhai, China; ^2^ Department of Hematology, Taizhou Hospital of Zhejiang Province Affiliated to Wenzhou Medical University, Linhai, China; ^3^ Department of Orthopedics, Taizhou Hospital of Zhejiang Province Affiliated to Wenzhou Medical University, Linhai, China; ^4^ Department of Neurosurgery, Taizhou Hospital of Zhejiang Province Affiliated to Wenzhou Medical University, Linhai, China

**Keywords:** healthcare workers, COVID-19, China, fatigue, pandemic evaluation

## Abstract

**Objectives:** To evaluate COVID-19 burnout syndrome among healthcare workers in Taizhou, China.

**Methods:** A total of 1,103 qualified healthcare workers in Taizhou were included in the study. The Maslach Burnout Inventory–General Survey (MBI-GS) was used to assess burnout syndrome.

**Results:** Among the healthcare workers surveyed, 25.9% experienced COVID-19 burnout syndrome, including 22.3% and 3.6% with mild and moderate burnout, respectively. Multivariate linear regression models revealed associations with emotional exhaustion among healthcare workers, as follows: occupation, education level and professional qualifications. Professional efficacy was impacted by the pandemic, as follows: sex and occupation. The following factors were associated with cynicism among healthcare workers: occupation and underlying disease. Occupation (medical technician vs. physician, *β* = −7.40, 95% confidence interval: −12.09 to −2.71, *p* = 0.002) was significantly related to MBI-GS scores after adjusting for confounding factors.

**Conclusion:** COVID-19 burnout syndrome was common among healthcare workers in Taizhou, China, and its impact was more burdensome to physicians.

## Introduction

The coronavirus disease 2019 (COVID-19) pandemic has been characterized by high levels of infectivity, asymptomatic infection, and early transmission (1, 2), and continues to be a public health emergency globally. Moreover, many countries are experiencing enormous pressure on their healthcare systems (3). The government of China has taken comprehensive action to contain the spread of the disease (4). However, the pandemic has altered the habits of healthcare workers as they had to adhere to more stringent cleaning routines and quarantine measures, implement the use of personal protective equipment, such as masks, face shields, gloves and gown, and separate patients with respiratory infections from others (5). Adhering to these strategies in practice can be time-consuming and sometimes difficult. Physical and emotional exhaustion associated with managing large numbers of patients and family members, the risk for nosocomial infections, and improving case identification, have rendered healthcare workers more vulnerable to the physical and psychological effects of the COVID-19 pandemic (6, 7).

Around September 2021, with the implementation of effective prevention and control measures, the COVID-19 pandemic in China entered a relatively stable period. However, a few cities in China continue to have local outbreaks due to various reasons, leading to unpredictable local lockdowns. Many places in China are simultaneously under lockdown. As the public’s patience with the COVID-19 pandemic wears thin, fatigue and numbness build, and clear signs of a new pandemic are rapidly emerging.

Frontline health professionals have been exposed to a higher risk for experiencing physical and mental health problems including depression, anxiety, distress, and symptoms of post-traumatic stress during the COVID-19 pandemic (8–10). Burnout syndrome is a biological symptom influencing the body and mind (11), a combination of high exhaustion and depersonalization, and low personal accomplishment (12). Burnout syndrome has profound effects on the mental health status of healthcare workers (13), it affected cognitive, behavioral, and psychomotor function, and could lead to delayed reactions, reduced motivation, and poor judgment (9). These negative impacts can lead to an increase in medical errors and/or treatment failure (10), a high burden on healthcare systems due to high rates of turnover and resignation among healthcare workers with poor mental health outcomes (14). The prevalence of burnout syndrome is highest among nurses, younger persons, and trainees (13). The most frequent risk factors associated with burnout syndrome among healthcare workers include stress, lack of family support, and organizational risk factors such as prolonged night shifts, length of experience, and exposure to traumatic events (13).

As such, there is an urgent need to review the problems encountered by health workers over the past year and take early preventive measures to prevent burnout from worsening. The pandemic will not subside anytime soon, and healthcare workers remain directly involved in responding to this emergency and are subject to significant psychological stress related to the uncertain duration of the crisis, the lack of proven therapies and/or vaccines, and the potential shortage of medical resources.

In this study, healthcare workers in Taizhou, Zhejiang Province, were selected as research subjects. This research mainly aimed to reveal the level of burnout of healthcare workers in a certain period of the pandemic and to define its personal predictors. It is also a timely investigation study to assess the burnout syndrome of medical staff in hospitals or other medical systems in medical emergency situations (such as COVID-19). This study highlights burnout syndrome in the context of long-term epidemiology, and includes various medical professionals, not just doctors and nurses.

## Methods

### Study Design and Participants

This study was conducted within the context of an ongoing project aiming to assess the impact of COVID-19 among hospital personnel in Taizhou, China. We organized a population-based, anonymous, cross-sectional online survey on the WeChat-incorporated Wen-Juan Xing platform (Changsha Ranxing Information Technology Co., Ltd., Hunan, China). The target population was healthcare workers at a medical center in Taizhou, China. Based on the convenience sampling method, data collection was performed in September 2021. Participants of this study were consistent with those included in previous research (15). The invitation to participate and the link to the online questionnaire were posted on the hospital’s website and sent to all hospital staff (*n* = 4,191) from a list *via* the WeChat Group, with 1,103 hospital staff accepting the survey invitation, the response rate was 26.3%. This study was exempt from informed consent and was approved by the Ethics Committee of Taizhou Hospital, Zhejiang Province, China (Approval number: K20210823). The survey was anonymous and the confidentiality of the information collected was guaranteed.

### Measurement Instruments

#### Demographic Data

The survey included general demographic data and one questionnaire. General demographic data included sex, age, occupational class, professional qualifications, education level, and underlying diseases (Table 1).

**TABLE 1:** Measurement of variables (Taizhou, China, 2021).

Variables	Definition	Coding
Age	40 is the age to be a mature healthcare worker in China. So we take 40 years old as an age node	0, <40 years
		1, ≥40 years
Education level	The education level refers to a person’s highest degree	0, College and below
		1, Undergraduate and above
Professional qualifications	In China, professional qualifications are divided into junior, intermediate and senior levels through a strict assessment system	0, Primary and below
		1, Intermediate and Senior
Underlying disease	Underlying diseases refer to chronic metabolic diseases, which require long-term treatment with drugs. It refer to hypertension, hyperlipidemia and diabetes in the paper	0, no hypertension, hyperlipidemia and diabetes
		1, with hypertension, or hyperlipidemia, or diabetes

#### Maslach Burnout Inventory—General Survey

Burnout was measured using the Maslach Burnout Inventory- General Survey (MBI-GS) (16), which was previously translated into Chinese and has demonstrated good reliability and validity in a Chinese sample. The Maslach Burnout Inventory (MBI) is considered the “gold standard” for measuring job burnout (17). It consists of 15 items rated on a Likert scale from 0 to 6 points. The MBI-GS has three subscales: exhaustion (5 items); cynicism (4 items); and professional efficacy (6 items) (18). The score for each domain is obtained by summing the scores of all items contained in each domain, and the total score for burnout for each domain is obtained by summing the scores of all domains. Add up the scores and divide by 15 to find the average score and multiply by 20 (which translates to 100 standard points). No burnout refers to score under 50 points; mild burnout refers to score 50 to 75 points; moderate burnout refers to score over 75 points (18).

### Statistical Analysis

Some continuous data, such as age, were converted to categorical data, which are expressed as count and frequency distributions and were subjected to univariate analysis to identify useful factors. Variables that were significant at the *p* < 0.05 level in univariate analysis were then selected for multivariate linear regression analysis. The associations of potential factors, such as sex, age, occupation, education level, professional qualifications, and underlying disease were assessed using *p* values, the standard regression coefficient (β) and corresponding 95% confidence interval (CI). All data were analyzed using SPSS version 26.0 (IBM Corporation, Armonk, NY, United States). Differences with *p* < 0.05 were considered to be statistically significant.

## Results

A total of 286 (25.9%) of the 1,103 healthcare workers surveyed reported experiencing COVID-19 burnout syndrome, including 246 (22.3%) and 40 (3.6%) with mild and moderate burnout, respectively. Personal and job characteristics of the participants are summarized in Table 2. The study cohort comprised 934 females and 169 males, 63.6% were nurses, 16.7% were physicians, 11.8% were medical technicians, and 7.9% held administration or part-time positions.

**TABLE 2:** Personal and job characteristics of participants who completed the MBI-GS (*n* = 1,103) (Taizhou, China, 2021).

Variables	n	%
Sex		
Male	169	15.3
Female	934	84.7
Age		
<40	832	75.4
≥40	271	24.6
Occupation		
Physician	184	16.7
Nurse	702	63.6
Medical technician	130	11.8
Administration or Part-time position	87	7.9
Education level		
College and below	243	22.0
Undergraduate and above	860	78.0
Professional qualifications		
Primary and below	593	53.8
Intermediate and Senior	510	46.2
Underlying disease		
No	971	88.0
Yes	132	12.0

Of the participants’ professional qualifications, 53.8% were primary and below, 46.2% were intermediate and senior. Moreover, 78.0% of the participants had undergraduate and above education and 88.0% did not have an underlying disease.

Data reported in Table 3 reveals that emotional exhaustion among healthcare workers was related to occupation (*p* < 0.001), education level (*p* < 0.001), and professional qualifications (*p* < 0.001). Professional efficacy was related to gender (*p* < 0.001), occupation (*p* < 0.001), education level (*p* = 0.001), and professional qualifications (*p* = 0.001). Cynicism was related to occupation (*p* < 0.001) and underlying disease(s) (*p* = 0.046). The total post-analysis Cronbach’s alpha coefficient for this study was 0.84, and 0.95, 0.95, and 0.95, for the three domains, respectively.

**TABLE 3:** Personal and job-related characteristics across the three burn-out domains and MBI-GS (*n* = 1,103) (Taizhou, China, 2021).

Variables	Categories	Emotional exhaustion mean ± SD	*P*	Professional efficacy mean ± SD	*P*	Cynicism mean ± SD	*P*	MBI-GS mean ± SD	*P*
Total	**--**	8.81 ± 6.95	**--**	4.29 ± 4.71	**--**	12.28 ± 9.28	**--**	33.83 ± 19.85	**--**
Gender	Men	9.38 ± 6.84	0.24	5.68 ± 4.78	<0.001	13.30 ± 8.88	0.12	37.80 ± 19.67	0.005
	Women	8.70 ± 6.96		4.04 ± 4.66		12.10 ± 9.35		33.11 ± 19.81	
Age (yrs)	<40	8.65 ± 6.81	0.20	4.21 ± 4.58	0.31	12.41 ± 9.25	0.43	33.70 ± 19.76	0.67
	≥40	9.27 ± 7.33		4.54 ± 5.10		11.90 ± 9.40		34.27 ± 20.14	
Occupation	Physician	9.40 ± 6.02	<0.001	5.90 ± 4.62	<0.001	13.98 ± 7.78	<0.001	39.03 ± 18.84	<0.001
	Nurse	9.26 ± 7.17		4.11 ± 4.80		11.42 ± 9.41		33.04 ± 20.40	
	Medical technician	6.28 ± 6.52		3.52 ± 4.13		12.46 ± 9.31		29.68 ± 18.68	
	Administration or Part-time position	7.67 ± 6.67		3.48 ± 4.29		15.39 ± 10.10		35.39 ± 16.90	
Education level	College and below	7.03 ± 6.83	<0.001	3.44 ± 4.77	0.001	13.05 ± 10.44	0.14	31.36 ± 19.61	0.03
	Undergraduate and above	9.31 ± 6.90		4.53 ± 4.67		12.06 ± 8.92		34.53 ± 19.87	
Professional	Primary and below	7.82 ± 6.78	<0.001	3.86 ± 4.67	0.001	12.57 ± 9.59	0.27	32.33 ± 19.73	0.007
qualifications	Intermediate and Senior	9.96 ± 6.97		4.78 ± 4.72		11.95 ± 8.91		35.58 ± 19.87	
Underlying disease	No	8.82 ± 7.00	0.88	4.27 ± 4.66	0.75	12.49 ± 9.30	0.046	34.10 ± 19.76	0.22
	Yes	8.72 ± 6.64		4.41 ± 5.09		10.77 ± 9.07		31.86 ± 20.49	

The MBI-GS score was associated with gender (*p* = 0.005), occupation (*p* < 0.001), education level (*p* = 0.03), and professional qualifications (*p* = 0.007).

Results of the multivariate linear regression models are summarized in Table 4.

**TABLE 4:** Multivariate linear regression models for the three burn-out domains and MBI-GS (*n* = 1,103) (Taizhou, China, 2021).

Variables	Emotional exhaustion				Professional efficacy				Cynicism				MBI-GS			
	β	SE	95%CI	*P*	β	SE	95%CI	*P*	β	SE	95%CI	*P*	β	SE	95%CI	*P*
Intercept	6.98	0.74	5.53, 8.43	<0.001	4.55	0.57	3.42, 5.67	<0.001	14.37	0.70	13.00, 15.73	<0.001	34.53	2.43	29.77, 39.29	<0.001
Gender	—	—	—	—	1.06	0.48	0.12, 2.00	0.03	—	—	—	—	2.76	2.03	−1.22, 6.73	0.17
Men vs. Women																
Occupation	0.54	0.58	−0.60, 1.67	0.35	−0.99	0.48	−1.93, −0.06	0.04	−2.74	0.76	−4.24, −1.24	<0.001	−3.62	2.02	−7.58, 0.34	0.07
Nurse vs. Physician																
Medical technician vs. Physician	−2.16	0.80	−3.74, −0.59	0.007	−1.78	0.56	−2.88, −0.67	0.002	−1.68	1.05	−3.75, 0.38	0.11	−7.40	2.39	−12.09, −2.71	0.002
Administration or Part-time position vs. Physician	−0.20	0.95	−2.07, 1.67	0.83	−1.46	0.69	−2.80, −0.11	0.03	1.28	1.20	−1.06, 3.63	0.28	−0.52	2.90	−6.22, 5.17	0.86
Education level Undergraduate and above vs. College and below	1.29	0.57	0.17, 2.40	0.02	0.39	0.39	−0.37, 1.16	0.31	—	—	—	—	1.51	1.64	−1.72, 4.73	0.36
Professional	1.62	0.45	0.74, 2.51	<0.001	0.49	0.31	−0.12, 1.09	0.11	—	—	—	—	2.00	1.30	−0.56, 4.56	0.13
qualifications																
Intermediate and Senior vs. Primary and below																
Underlying disease	—	—	—	—	—	—	—	—	−2.03	0.86	−3.71, −0.35	0.02	—	—	—	—
yes vs. no																
Adjusted R^2^ = 0.05					Adjusted R^2^ = 0.03				Adjusted R^2^ = 0.02				Adjusted R^2^ = 0.02			

Regression analysis revealed the following associations between burnout syndrome and emotional exhaustion among healthcare workers: medical technician vs. physician (*β* −2.16 [95% CI −3.74 to −0.59]; *p* = 0.007); undergraduate and above vs. college and below (*β* 1.29 [95% CI 0.17 to 2.40]; *p* = 0.02); intermediate and senior vs. primary and below (*β* 1.62 [95% CI 0.74 to 2.51]; *p* < 0.001). Burnout syndrome affected professional efficacy among healthcare workers as follows: male vs. female (*β* 1.06 [95% CI 0.12 to 2.00]; *p* = 0.03); nurse vs. physician (*β* −0.99 [95% CI −1.93 to −0.06]; *p* = 0.04); medical technician vs. physician (*β* −1.78 [95% CI −2.88 to −0.67]; *p* = 0.002); administration or part-time position vs. physician (*β* −1.46 [95% CI −2.80 to −0.11]; *p* = 0.03). Analysis of healthcare worker cynicism revealed the following: nurses vs. physicians (*β* −2.74 [95% CI −4.24 to −1.24]; *p* < 0.001); and underlying disease (yes vs. no) (*β* −2.03 [95% CI −3.71 to −0.35]; *p* = 0.02). MBI-GS scores demonstrated the following association: medical technician vs. physician (*β* −7.40 [95% CI −12.09 to −2.71]; *p* = 0.002).

## Discussion

The present study yielded several main findings. First, 25.9% of healthcare workers experienced burnout syndrome. Second, emotional exhaustion among participants was associated with occupation, education level, and professional qualifications. Third, professional efficacy of the participants was associated with gender and occupation. Fourth, cynicism among participants was associated with occupation and underlying disease. Fifth, physicians had higher MBI-GS scores than other medical professions.

Healthcare workers are susceptible to burnout syndrome due to the high intensity of their work, heavy responsibilities, and high occupational risks. Serious job burnout will threaten the physical and mental health of healthcare workers, affect the quality of medical and health services, and negatively impact the harmonious and stable development of society.

Burnout among healthcare workers has been exacerbated by the COVID-19 pandemic (19). Before January 2021, a systematic review and meta-analysis provided a complete assessment of the prevalence of fatigue across various medical staff, and found that the pooled overall prevalence of burnout was 52% (19). Our research revealed that 25.9% of healthcare workers experienced burnout in Taizhou, China, in September 2021. As COVID-19 continues to evolve, the incidence of burnout may also be changing as the pandemic is effectively brought under control.

Before COVID-19, the number of patients per nurse in our hospital was less than 8, and the number of doctors per nurse was more than 1:1.15, in accordance with China’s standard for Class A tertiary hospitals (20, 21). After the COVID-19 pandemic, some medical staff must participate in additional work on COVID-19 prevention and control (such as nucleic acid sampling and other events for COVID-19 prevention and control), resulting in an increase in the *per capita* workload. Some nurses may have to care for more than 8 patients on duty. Moreover, with the progress of COVID-19 and the repeated changes of various COVID-19 epidemics in 2020 and 2021, the burnout syndrome among healthcare workers had accumulated to a new level by September 2021.

In studying job burnout, emotional exhaustion mainly refers to the exhaustion of personal emotional resources, which is mainly manifested as work pressure, lack of motivation, fatigue, and continuous tension and frustration with work (22). With the spread of COVID-19, the increased workload has created greater stress and emotional problems for healthcare workers as they become more prone to emotional exhaustion (23–25). This is especially true among frontline healthcare workers (25), such as nurses and physicians. In our study, we found that medical technician vs. physician, undergraduate and above vs. college and below education level, intermediate and senior vs. primary and below were associated with emotional exhaustion among healthcare workers. Therefore, it is reasonable to suggest that, to control the emotional exhaustion of hospital staff, we should focus on physicians, staff with low educational levels, and those with primary and below professional qualifications.

It is well known that professional efficacy is very important in the field of medicine. Chinese healthcare workers exert more external effort to maintaining high levels of professional efficacy (26), which appears to be a positive coping mechanism in the short term but can lead to energy exhaustion and disengagement over time. A previous study reported that individuals who are constantly tired and cynical eventually experience professional inefficacy (27). Burnout is different between the sexes and the determinants of burnout also differ between the sexes (28). An interesting finding in our research was that professional efficacy was associated with sex and occupation: male vs. female, nurse vs. physician, medical technician vs. physician, administration or part-time position vs. physician. In other words, compared with females, males tended to have low professional efficacy compared with other medical personnel, and physicians were prone to the problem of low professional efficacy. We speculate that to address the problem of low professional efficacy, more attention should be devoted to male physicians.

Cynicism is another symptom of burnout and can fuel the intent to leave work (29). Males have reported higher cynicism than females (30). However, in our study, there was no significant difference in cynicism between males and females. Healthcare workers are mildly cynical toward their institutions and nurses experience organizational cynicism at an intermediate level (29). In our study, we found that cynicism among participants was associated with occupation and underlying disease. Compared with physicians, nurses reported higher levels of cynicism, which may be caused by lower pay, longer work hours, and not being valued or appreciated (29).

Nurses are particularly prone to burnout, and the risk is even higher for those working in emergency departments (31). Being a nurse increases the risk for burnout compared with physicians (32). We found that physician experienced higher level of burnout than medical technician according to MBI-GS score. There were no statistically significant differences in burnout syndrome between nurses and physicians, or between administration or part-time position and physicians, which contradicts some previous studies (31, 32). This may be because physicians have higher job expectations, and when those expectations are not met, satisfaction declines and emotional exhaustion sets in, leading to job burnout. It may also be due to the need for COVID-19 prevention and control, leading to extended work hours and increased complications of routine work processes, resulting in low work efficiency and resulting in job burnout. Of course, the clinical work of physicians and nurses is interwoven, with nurses assuming more responsibility for infection prevention and control and relevant vaccinations, nucleic acid monitoring, and sampling work, thus leading to clinical nursing shortages, which may require physicians to take on more clinical work, which, in turn increases the incidence of job fatigue among physicians.

Research has also shown that remuneration is related to job burnout and that improving the remuneration system may reduce the occurrence of burnout among medical staff (33); however, the overall salary level of physicians in China deviates from the labor value. Preliminary findings of this study found that only approximately one-half of healthcare workers were willing to pay for a booster dose of inactivated severe acute respiratory syndrome coronavirus 2 (i.e., SARS-CoV-2) vaccine in China, with most only willing to pay <100 CHY ($15.04 USD) (15). This is significantly lower than that of healthcare workers from some other countries with comparable economic levels (34–36). This is a reflection of the salaries of physicians in China. Combined with the study by Zhu (33), we have reason to believe that the deviation of the overall salary level of physicians from the labor value may be related to the high incidence of job fatigue among Chinese physicians.

### Clinical Perspectives

• The present study aimed to evaluate COVID-19 burnout syndrome among healthcare workers in Taizhou, China.
• To minimize the occurrence of job burnout, compared with other hospital staff, hospital management should devote more attention to physicians. Meanwhile, intervention can be implemented from multiple dimensions. To control emotional exhaustion, we should focus on physicians, personnel with low educational levels, and staff with primary and below professional qualifications, and provide them with more opportunities to improve their education and more opportunities for promotion. Compared with physicians, nurses exhibited higher levels of cynicism; as such, hospital management should enhance the sense of accomplishment by promoting nurses’ participation in decision-making, especially in demanding situations, and improving their sense of control over their own schedules and tasks. In addition, the hospital management department should hire more staff to reduce the non-clinical task burden of frontline medical staff, and limit excessive workload by arranging rest time.
• Hospitals should devote more attention to groups at high risk for job burnout, improve the salary system, and reasonably arrange the working hours of physicians in efforts to relieve burnout.

### Methodological Considerations

#### Strengths

This study has some strengths. First, it is a timely investigation to assess the burnout syndrome of medical staff in hospitals or other medical systems under medical emergency situations, rather than a study solely targeted at the COVID-19 period in China. Second, this study highlights the burnout syndrome in the context of long-term epidemiology. Third, the study included various medical professionals, not just doctors or nurses.

#### Limitations

Some limitations of this study should be addressed. First, its cross-sectional design does not enable the determination of causal relationships. It also prevented us from studying variations in burnout among healthcare workers throughout the COVID-19 period. Second, because the research participants all belonged to a single medical center in a single region, the sample may not fully or necessarily represent all medical workers in China. Third, the online approach to data collection is a limitation that may lead to excessive reporting the epidemic fatigue of COVID-19 among healthcare workers in Taizhou. Fourth, our questionnaire did not account for all possible risk factors related to job burnout of medical staff, such as the number of night shifts, working hours, and salary; as such, the research results may be biased. Fifth, the MBI-HSS may be more suitable for the assessment of burnout among professional service personnel, and the use of MBI-GS may cause some research bias. Sixth, we did not solve the problem of interaction between job demands and the demographic characteristics and occupations of the participants. Finally, because China has relaxed its epidemic prevention and control policies, the burnout of different medical staff may have changed dramatically.

### Conclusion

Burnout syndrome was common among healthcare workers during the COVID-19 pandemic in Taizhou, China, and its impact was more burdensome to physicians.
